# The silent and apparent neurological injury in transcatheter aortic valve implantation study (SANITY): concept, design and rationale

**DOI:** 10.1186/1471-2261-14-45

**Published:** 2014-04-05

**Authors:** Jonathon P Fanning, Allan J Wesley, David G Platts, Darren L Walters, Eamonn M Eeles, Michael Seco, Oystein Tronstad, Wendy Strugnell, Adrian G Barnett, Andrew J Clarke, Judith Bellapart, Michael P Vallely, Peter J Tesar, John F Fraser

**Affiliations:** 1 School of Medicine, The University of Queensland, Brisbane, Queensland, Australia; 2 Critical Care Research Group (CCRG), The Prince Charles Hospital, Rode Road, Chermside, Brisbane, Queensland 4032, Australia; 3 The Heart and Lung Institute, The Prince Charles Hospital, Brisbane, Queensland, Australia; 4 Department of Medical Imaging, The Prince Charles Hospital, Brisbane, Queensland, Australia; 5 School of Medicine, The University of Sydney, Sydney, NSW, Australia; 6 The Baird Institute, Sydney, NSW, Australia; 7 Department of Internal Medicine, The Prince Charles Hospital, Brisbane, Queensland, Australia; 8 Department of Cardiothoracic Surgery, The Prince Charles Hospital, Brisbane, Queensland, Australia; 9 Department of Cardiology, The Prince Charles Hospital, Brisbane, Queensland, Australia; 10 School of Public Health and Social Work, Queensland University of Technology, Brisbane, Queensland, Australia; 11 The Royal Prince Alfred, Sydney, NSW, Australia; 12 Macquarie University, Sydney, NSW, Australia; 13 Adult Intensive Care Unit, The Prince Charles Hospital, Brisbane, Queensland, Australia

**Keywords:** Aortic valve stenosis, Heart valve prosthesis implantation, Cerebrovascular disorders, Stroke, Embolism and thrombosis

## Abstract

**Background:**

The incidence of clinically apparent stroke in transcatheter aortic valve implantation (TAVI) exceeds that of any other procedure performed by interventional cardiologists and, in the index admission, occurs more than twice as frequently with TAVI than with surgical aortic valve replacement (SAVR). However, this represents only a small component of the vast burden of neurological injury that occurs during TAVI, with recent evidence suggesting that many strokes are clinically silent or only subtly apparent. Additionally, insult may manifest as slight neurocognitive dysfunction rather than overt neurological deficits. Characterisation of the incidence and underlying aetiology of these neurological events may lead to identification of currently unrecognised neuroprotective strategies.

**Methods:**

The *Silent and Apparent Neurological Injury in TAVI* (SANITY) Study is a prospective, multicentre, observational study comparing the incidence of neurological injury after TAVI versus SAVR. It introduces an intensive, standardised, formal neurologic and neurocognitive disease assessment for all aortic valve recipients, regardless of intervention (SAVR, TAVI), valve-type (bioprosthetic, Edwards SAPIEN-XT) or access route (sternotomy, transfemoral, transapical or transaortic). Comprehensive monitoring of neurological insult will also be recorded to more fully define and compare the neurological burden of the procedures and identify targets for harm minimisation strategies.

**Discussion:**

The SANITY study undertakes the most rigorous assessment of neurological injury reported in the literature to date. It attempts to accurately characterise the insult and sustained injury associated with both TAVI and SAVR in an attempt to advance understanding of this complication and associations thus allowing for improved patient selection and procedural modification.

## Background

**Figure 1:**
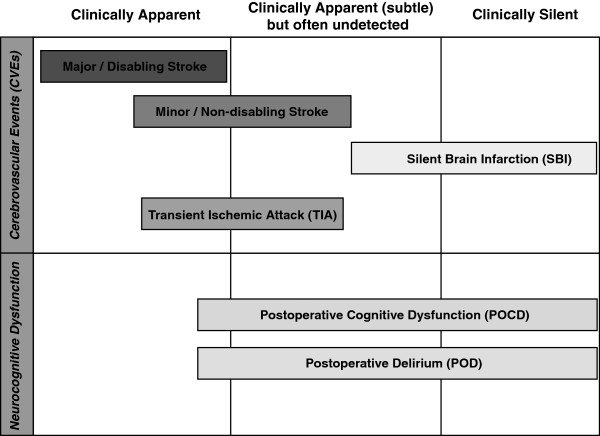
Schema of neurological injury in TAVI.

Emerging data now supports transcatheter aortic valve implantation (TAVI) as an acceptable strategy in the management of severe aortic stenosis (AS) amongst patients deemed ineligible or too high-risk for SAVR. Despite the technique’s success, the risk of neurological insult and injury associated with TAVI has raised concerns [1]. This may take the form of either cerebrovascular events (CVEs) – including major/disabling stroke, minor/non-disabling stroke, transient ischemic attacks (TIAs) or silent brain infarcts; or neurocognitive dysfunction - especially post-operative cognitive dysfunction (POCD) and post-operative delirium (POD). Such events may be overt, subtle, or clinically silent (Figure 1). While overt stroke rates have been reported in most safety and efficacy studies/registries, subtle and silent neurological events have been unreliably detected due to limitations of assessment tools and prevailing clinical approaches. Additionally, although emboli are thought to be the major cause especially of early neurological injury the underlying insults behind both types of injury remain poorly characterised.

### Clinically apparent cerebrovascular events

Strokes/TIAs (as defined in Table 1) are more common post-TAVI than after alternative management strategies, with the highest reported incidence for any cardiac procedure [2]. Thirty-day stroke rates of 3.3%, 2.4% and 1–2% have been reported for populations undergoing TAVI, isolated SAVR, and balloon valvuloplasty, respectively [3-5]. For all three interventions, this risk is highest within the first 24 hours, suggesting an early hazard phase immediately post-procedure [6,7]. This early hazard phase is considered 'procedurally-related’ and represents the period during which TAVI recipients are at increased risk. Improved understanding of events during this period therefore promises the greatest potential for optimisation of TAVI and as such is the focus of the SANITY Study.

**Table 1:** Classification and definitions of neurological injury/impairment

**A. Cerebrovascular events**[17]	
**Silent**	Cerebral infarcts that are observed on magnetic resonance imaging (MRI) scans in the absence of any corresponding, clinically apparent cerebrovascular ischaemic event.
**Clinically apparent**	Acute episode of a focal or global neurological deficit with at least one of the following: change in the level of consciousness, hemiplegia, hemiparesis, numbness, or sensory loss affecting one side of the body, dysphasia or aphasia, hemianopia, amaurosis fugax, or other neurological signs or symptoms consistent with stroke.
**a. Stroke**	Duration of a focal or global neurological deficit ≥ 24 hours; OR <24 hours if available neuroimaging documents a new hemorrhage or infarct; OR the neurological deficit results in death.
***Aetiology***	***i. *Ischemic:** an acute episode of focal cerebral, spinal or retinal dysfunction caused by infarction of the central nervous system tissue.
	**ii. Haemorrhagic:** an acute episode of focal or global cerebral or spinal dysfunction caused by intraparenchymal, intraventricular, or subarachnoid haemorrhage.
	**iii. Undetermined:** insufficient information to allow categorization as ischemic or haemorrhagic.
***Severity***	**i. Disabling stroke (Major):** an mRS of 2 or more at 90 days and an increase in at least one mRS category from an individuals pre-stroke baseline.
	**ii. Non-disabling stroke (Minor):** an mRS score of <2 at 90 days or one that does not result in an increase in at least one mRS category from an individual’s pre-stroke baseline.
	**b. TIA:**
**b. TIA**	Duration of a focal or global neurological deficit <24 hours, any available neuroimaging does not demonstrate a new haemorrhage or infarct.
***Qualifiers***	• Exclusion of non-stroke causes for clinical presentation
	o e.g. brain tumour, trauma, infection, hypoglycaemia, peripheral lesion, pharmacological influences etc.
	• Determined by or in conjunction with the designated internal medicine specialist or neurologist.
	• Diagnosis confirmed by at least one of the following:
	• Neuroimaging procedure (CT scan or MRI brain) and/or
	• Neurologist or neurosurgical specialist.
**B. Neurocognitive impairment**	
**a. POCD**	**Definition:** Deterioration of intellectual function presenting as impaired memory or concentration presenting with temporal association to surgery.
**b. POD**	**Definition:** An acute disturbance of consciousness and a change in cognition with tendency to fluctuate during the course of the day and occurring in patients without some other identifiable aetiology and following normal emergence from anaesthesia.
***Qualifiers***	
	• In conjunction with CAM and MoCA assessment tools

Adapted from Kappetein *et al.*[17] with permission of the publisher.

### Silent brain infarction

In neuroimaging studies, clinically apparent strokes account for only a minority of CVEs. Diffusion-weighted magnetic resonance imaging (DWI) studies have revealed the average incidence of new ischemic lesions as 75% post-TAVI, and as high as 93% [8,9]. Comparatively, only 40–50% are evident on DWI studies following SAVR, and 22% following retrograde catheterisation of the aortic valve in AS [8,10]. Understanding of these clinically silent CVEs is more limited than for clinically apparent strokes, with only a few published MRI-centred studies investigating their occurrence post-TAVI, amounting to fewer than 200 patients. The clinical relevance of this burden of infarct remains unknown, though has been suggested to correlate with increased risk of post-operative cognitive dysfunction (POCD) or post-operative delirium (POD) [11,12]. Furthermore, DWI has recently been recommended as a useful surrogate endpoint for quantifying neurological injury due to the relative rarity of clinically apparent neurological injury [13].

### Neurocognitive impairment

Investigation into neurological injury post-TAVI has almost exclusively focused on CVEs, with little attention given to neurocognitive/neuropsychological disturbances. This reflects both the common occurrence and uncertain consequences of such events and the difficulties of their clinical detection. Two entities of particular interest are POD and POCD, which are outlined in Table 1. Though clinical experience suggests that these are common entities post-cardiovascular intervention, studies geared towards their detection following TAVI are sparse.

### Neurological insult

The underlying neurological insult resulting in injury is thought to be primarily an embolic phenomenon [2]. Transcranial Doppler (TCD) studies have demonstrated microemboli at all stages of the TAVI procedure, with cumulative embolic loads exceeding 500 high-intensity signals (HITS, a validated surrogate of emboli) reported, especially during stages of aortic valve manipulation/TAVI deployment. A recent study employing embolic protection devices has demonstrated that a large number of such emboli are particulate in nature and include acute and chronic thrombus, atheromatous, calcific and valve/vascular tissue [14].

In addition to emboli, cerebral hypoperfusion is also likely a significant contributory factor, both in precipitating ischemia and magnifying the effects of microemboli [15]. Indeed, a pilot study employing cerebral oximetry has confirmed statistically significant reductions in cerebral oxygen saturations most notably during periods of rapid ventricular pacing during valvuloplasty and valve deployment [16].

Generally, the data used to quantify and characterise the aetiology of the neurological insult is based on a limited number of small studies. Furthermore, no studies to date have correlated neurological insult detected by intraoperative monitoring and MRI evidence of neurological injury within the same cohort of patients.

### Area of need

Clearly, current understanding of the nature and character of both the neurologic injury and the underlying insult associated with TAVI is incomplete. The paucity of data is the consequence of studies not specifically geared towards sensitive neurological insult/injury detection and the significant functional benefits of TAVI in patients who are ineligible or at high-risk for SAVR. However, the nature of neurological injury becomes of increasing interest as refinement of TAVI continues with the expectation that complications can and should be minimised. Indeed, the greatest barrier to TAVI extending into lower surgical risk and younger populations remain the risk of neurological injury.

### Objectives

The SANITY study (ACTRN12613000083796) aims to provide the most comprehensive characterisation to date of neurological insult and injury - both clinically apparent and silent – post-TAVI and SAVR. These encompass pre-existing patient factors, and both intra- and post-operative factors thought to influence neurological insult and injury during aortic valve interventions. It is hoped that improved understanding of these associations will permit detailed characterisation of neurological injury during TAVI compared to the gold-standard management of SAVR, and allow for the identification of predictors of such injury and previously unrecognised neuroprotective strategies.

Specifically, the objectives are:

*Primary Objectives:* Characterise neurological injury (MRI evidence of new ischaemic lesions) during the TAVI procedure compared with SAVR

Secondary Objectives:

1. Define the association between incidence of neurological insult (intraprocedural monitoring) and injury with:

– Procedure (TAVI, SAVR)

– TAVI access (transfemoral, transapical, transaortic)

2. Define the association between incidence of neurological insult, radiological evidence of injury and:

– Clinically apparent neurological injury

– New onset atrial fibrillation

– Neurocognitive dysfunction

– Degree of vascular/aortic valve calcification

– Rapid ventricular pacing and cerebral desaturation

– Serological markers of neurological injury (S100B and GFAP)

3. Validate GFAP as a serological marker of neurological injury in cardiovascular intervention

### Hypotheses

Based on the current concept of neurological insult and extrapolation of procedural risk factors for embolisation, it is hypothesised that neurological injury is less pronounced with:

1. SAVR than TAVI, due to the removal of the valvular source of embolisation, which in TAVI persists, pushed against the wall of the aorta and potentially acting as a nidus for calcific emboli and altered rheology, promoting thrombosis.

2. A transapical/transaortic than transfemoral approach. Transapical/transaortic routes minimise the risk of disrupting calcific plaques especially in the aortic arch, an inherent risk with femoral access.

## Methods

### Study design

This is a prospective, multicentre, non-randomised and observational study comparing the incidence of neurological injury associated with TAVI of the Edwards SAPIEN-XT (Edwards LifeSciences Irvine, CA, USA) under general anaesthetic versus bioprosthetic SAVR. Ethics approval has been obtained from the Human Research Ethics Committee of the Prince Charles Hospital, Brisbane, Australia (HREC/12/QPCH/291) and informed consent will be obtained from all participants prior to enrolment.

**Figure 2:**
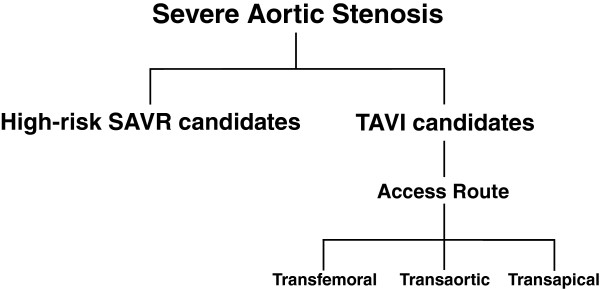
SANITY study design.

Regularly held 'Heart Team’ meetings - comprising of multiple disciplines including echocardiologists, interventional cardiologists, nurses and cardiothoracic surgeons – will assess high-risk patients with severe AS. Here, patients will be allocated to the intervention clinically indicated. Pending informed consent, such patients will be consecutively recruited into the SANITY study, resulting in cohort grouping as outlined in Figure 2.

### Patient population

Thorough pre-, intra- and post-procedural assessments allow results to be adjusted for baseline status, thus permitting an inclusive approach to enrolment. As such, only patients unable to participate in aspects of assessment or enrolled in other studies (Table 2) will be excluded.

**Table 2:** Eligibility criteria

**Inclusion criteria**	**Exclusion criteria**
I. Informed consent for participation	I. Lacks capacity to consent for him or herself
II. Severe aortic stenosis	
i. AVA <0.8 cm^2^	II. Pre-existing neurological impairment
ii. Mean aortic valve gradient >40 mmHg	i. Modified rankin score ≥ 3 (i.e. moderate disability; requiring some help, but able to walk without assistance)
iii. Peak jet velocity >4 m/s	
III. Planned TAVI or SAVR	
IV. High-surgical-risk	III. Contraindication to MRI (including incompatible metallic prosthesis/foreign body, inability to lie flat, claustrophobia requiring sedation)
i. STS score >8%	
ii. Logistic EuroSCORE >20%	
iii. Logistic EuroSCORE II >10%	IV. Non or poor English-speaking due to nature of and unknown validity in such populations cognitive testing
	V. Previous aortic valve repair/replacement
	VI. Coronary artery disease requiring revascularisation (including patients undergoing combined AVR and CABG)

### Data collection

**Figure 3:**
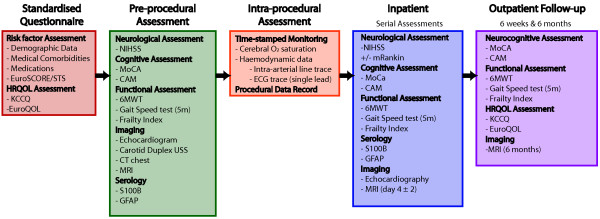
Overview of SANITY assessments.

The complete assessment regime and timing of each component can be found in Figure 3. Where relevant, this regime was designed to be consistent with the assessments and endpoints recommended by the *Valve Academic Research Consortium* updated standardised endpoints (VARC-2) [17]. A multi-disciplinary approach has been adopted to ensure that relevant experts address each assessment domain.

#### *Medical*/*medication history*

Qualified medical or nursing personnel will administer a detailed, standardised questionnaire, either at the time of consent or, in cases of telephone consent, at first subsequent contact. This is aimed at identifying all pre-existing cerebrovascular and neurocognitive risk factors and assigning formal surgical-risk scores using the *Society for Thoracic Surgeons Predicted Risk of Mortality* (STS-PROM) scale and *European System for Cardiac Operative Risk Evaluation* (EuroSCORE) II. At each contact specific questioning and chart review will target transient ischemic events that might otherwise be missed using the neurological assessment protocol outlined below.

### Neurological and cognitive assessment

There are no standards or recommendations guiding neurocognitive assessment in the TAVI population. Cognitive function will primarily be assessed with application of the Montreal Cognitive Assessment (MoCA). This tool is generally considered more sensitive for vascular cognitive impairment than the mini-mental state examination (MMSE) due to the inclusion of a thorough executive function assessment [18]. Additionally, as appropriate, the Confusion Assessment Method (CAM) or the intensive care unit (ICU) equivalent – CAM-ICU, will be performed for the detection of delirium [19]. These tests will be administered prior to the index procedure and at day 3, 6 weeks and 6 months post-index procedure.

### Clinical neurological assessment

The National Institutes of Health Stroke Scale (NIHSS) is a widely validated tool, which facilitates the standardised objective quantification of impairment caused by stroke. Categorisation of stroke severity (no stroke, minor stroke, moderate stroke, moderate to severe stroke and severe stroke) is permitted, based on the overall score. Where stroke is identified (pre-existing or new), the modified Rankin Scale (mRS) will be applied to measure and monitor stroke-related disability. These assessments will be applied at the same time points as outlined for the cognitive assessment tools.

### Serological assessments

Numerous serological markers of neurological injury have been utilised previously in acute ischemic stroke (AIS), however few studies have evaluated their application specifically in patients undergoing cardiovascular interventions; thus, the ideal marker in this setting is unknown [20]. Following careful consideration of strengths and weaknesses of each (Additional file 1: Table S1), GFAP and S100B were selected for use in the SANITY study. The time points for testing will be pre-procedure and daily for four days post-procedure.

### Imaging assessments

#### Echocardiography

Echocardiography (either transoesophageal or transthoracic) will be performed on all patients prior to and following the aortic valve procedure for the purposes of grading aortic stenosis and valve function. The TAVI Echocardiographic Calcification Score (TAVI-ECS) will be applied [21]. Additionally, assessment for intra-cardiac thrombi and spontaneous echo contrast will be sought to aid assessment of pre-existing procoagulant state [22].

#### Carotid duplex ultrasound

It is feasible that carotid artery stenosis (CAS) may play a role, albeit currently undefined, in post-operative stroke [23]. As such, carotid duplex ultrasound will be performed on all patients at baseline to identify CAS within the common, internal and external carotid arteries. Stenosis and plaque burden will be graded, taking into consideration all information from B-mode, pulsed-wave and colour-flow Doppler.

#### CT Chest*/*vascular calcification score

Arterial calcification correlates with atherosclerotic plaque burden [24]. The Agaston Score Equivalent (ASE) is a widely used method of measuring the calcified plaque burden in coronary arteries using non contrast CT [25]. Thus, a low dose, non-contrast acquisition will form part of the CT angiographic assessment of the aorta and the iliofemoral access arteries as routinely performed for TAVI suitability assessment and planning. These images will be loaded into Syngo.Via (Siemens Healthcare, Munich, Germany) and Calcium Score/ ASEs of the aorta will be quantitatively calculated for each of the aortic vessel segments, including the aortic root and valve leaflets. The correlation between the vascular and aortic valve plaque burden and neurological insult can thus be determined.

#### Magnetic resonance imaging *(*MRI*)*

Recognised as the most sensitive technique for the detection of acute ischemic cerebral infarcts, DWI detects the restriction of water diffusion in cerebral tissue caused by hypoxic oedema within minutes of ischemia onset. Increased signal intensity on DWI is reliably Detectable 4 hours post-insult and persists for up to 10 days [26]. Lesions representing brain infarct can be quantified in number, size, and volume using this modality.

Susceptibility Weighted Imaging (SWI) is a relatively new MRI sequence which is highly sensitive to the local magnetic field inhomogeneity caused by paramagnetic substances including the haem group in haemoglobin. SWI is commonly used as an adjunct to DWI in the assessment of cerebral ischemia as it is extremely sensitive for detecting haemorrhagic transformation within regions of infarction; can demonstrate acute thromboemboli sufficient to occlude arteries and can detect micro-haemorrhage, suspected to originate from diapedesis of red blood cells across overtly permeable capillaries. Peak incidence of haemorrhagic transformation occurs between 48 hours and 5 days and can persist for years [27].

Patients will undergo a baseline MRI study <48 hours prior to intervention. This comprehensive study will include standard fast spin echo sequences, baseline DWI and SWI sequences and time of flight angiography of the circle of Willis to document pre-existing disease. At day 4 (±2) post-intervention, and following removal of temporary pacing wires where applicable, a limited study consisting of DWI, SWI, T2 Flair and T1-weighted imaging will be performed. This interval length allows for sufficient patient recovery post-procedure to safely undergo an MRI examination and allows for establishment of the MRI detectable changes and maximum evolution of the ischemic change [28]. This limited acquisition is performed in 15 min for increased patient tolerance. A further limited examination after 6 months will include DWI and SWI, demonstrating DWI resolution, infarct complications and finally accounting for all SWI lesions.

### Monitoring

#### Near infrared spectroscopic *(*NIRS*)* Cerebral Oximetry

Transcranial cerebral oximetry non-invasively monitors cerebral oxygen saturation (rSO_2_) in the frontal lobes of the brain and has been shown to correlate with cerebral venous oxygen saturation, which is traditionally considered the 'gold-standard’ for determining oxygen delivery/consumption [29]. Accepted thresholds for cerebral ischemia are a rSO_2_ of <50% or a decline of >20% [30]. This will be applied during the intra-operative period of the SANITY study with use of INVOS™ (Covidien, Boulder, CO) specifically to monitor the cerebral oxygen delivery compromise associated with the procedure, and in particular with rapid ventricular pacing [31,32].

#### Invasive blood pressure

Hemodynamic instability leading to systemic hypotension may impair cerebral perfusion pressure beyond auto-regulatory capacity, resulting in hypoperfusion [33]. While itself a cause of ischemia, low cerebral flow magnifies the effects of micro-emboli by impairing their clearance and permitting small emboli to lodge.

#### Telemetry

Intra-operative telemetry will be employed to measure the number and duration of rapid ventricular pacing episodes and to detect intra-operative rhythm abnormalities.

#### Quality of life

Quality of life (QoL) assessment during post-procedural follow-up is crucial to determining the clinical benefit of TAVI and fully appreciating the consequences of neurological and cognitive insult and injury. Recommendations of the VARC-2 necessitate both a health-specific measure and generalised measure of QoL [34]. Following careful consideration of each (Additional file 2: Table S2), the heart failure specific Kansas City Cardiomyopathy Questionnaire (KCCQ) [35], and the generic European Quality of Life instrument-5D (EQ-5D) [36] were selected. These will be performed prior to and 6 months following index procedure.

#### Functional outcome measures and frailty

Functional capacity will be measured objectively by the six-minute walk test (6MWT), conducted according to American Thoracic Society guidelines [37]. This simple, practical and inexpensive sub-maximal exercise test assesses functional capacity [38], is closely linked to activities of daily living and has been proposed as both a functional status indicator and an outcome measure [39]. Similarly, gait speed is an important indicator of frailty [40], with slow gait a strong predictor of adverse outcomes, including disability, high healthcare utilisation and mortality [41].

Frailty describes a vulnerability to adverse events that is associated with, but separate from chronological age [42]. Implicitly, all patients requiring TAVI as opposed to open aortic valve surgery must have frailty as a barrier to standard treatment. Frailty is therefore an important contributor to the choice of TAVI and a potential confounder regarding outcomes, including delirium [43]. The SANITY study will utilise the Deficit accumulation score, which provides a robust measure of frailty and an appreciation of those who are unable to complete performance based tests - the frailest frail [44].

### Endpoint definitions

**Figure 4:**
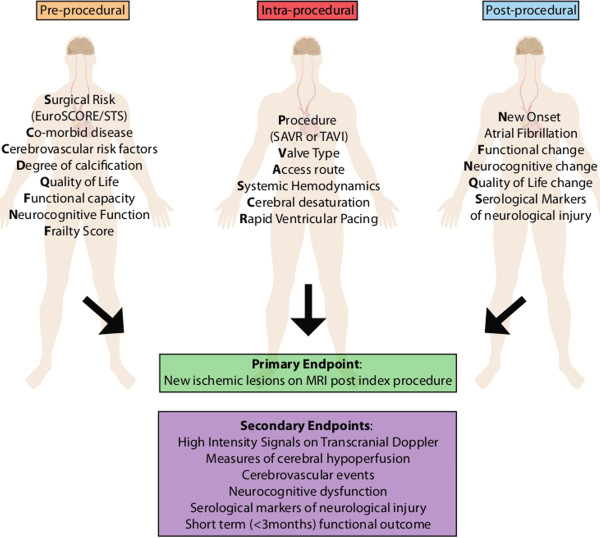
Overview of SANITY variables and endpoints.

The endpoints for the SANITY study are outlined in Figure 4. The primary endpoint focuses on the characterisation and quantification of the neurological injury as detected by new ischemic lesions on MRI. Secondary endpoints focus on the clinically apparent neurological injury sustained and markers of insult/injury.

### Statistical analysis

An estimated 100 patients will be recruited into this study, fifty in each of the two treatment arms (SAVR and TAVI) and at least 20 patients in each TAVI subgroup (transfemoral and transapical/transaortic). Fifty patients per group provides 90% power to detect differences in the incidence of new DWI lesions (primary endpoint) with two-sided statistical significance of 5%, assuming overall incidence estimates of 76% and 45% with TAVI and SAVR, respectively, as previously reported.

Multiple regression models will be used to adjust for potential confounders identified based upon clinical importance and statistical selection. The key output will be the estimated treatment difference and 95% confidence intervals for the primary group from the multiple regression models. Additionally, longitudinal analysis will be used to examine all outcomes with repeated data, again using multiple regression models. Treatment failure and withdrawal will be considered on an intention-to-treat basis, with the aim of providing a more realistic estimate of the difference between groups in clinical practice.

## Discussion

The SANITY Study offers the most comprehensive neurological/neurocognitive assessment of the TAVI and high-risk SAVR patient population to date. Additionally, well-established and recommended assessments are complemented by those that are novel and unique, thereby offering the potential of improved understanding of the interventions in question, correlation of risk factors and prognostic markers, and validation of assessment tools themselves. Such knowledge is vital to establishing both suitable assessment batteries and guidelines in this unique, high-risk group of patients, and to advancing strategies for neurological injury reduction.

### Limitations

Across all TAVI literature, the identification of risk-matched control groups has proven controversial. The validity of high-risk SAVR patients as a 'control group’ has previously been questioned, given that the level of health risk is a factor determining suitability for the procedure. Such difference also exists between access approaches, with transapical/transaortic typically considered a second line option where severe vascular disease excludes a transfemoral approach. Furthermore, these procedures differ vastly, most notably in the use of cardiopulmonary bypass, obscuring the exact cause of any difference identified between study groups. However, comparison between these two groups does offer clinical relevance as SAVR is the current 'gold-standard’ for eligible patients with AS and the challenge is in selecting the most appropriate procedure in patients who are considered suitable for both.

This study will be non-randomised and non-blinded. Though multiple regression analysis will be used to minimise confounding, this is not a substitute for blinding or randomisation, and unmeasured confounders may produce hidden bias.

Finally, a clinical follow-up duration of 6 months limits conclusions regarding delayed and late neurological injury. However, such a timeframe can be justified, given that DWI findings resolve within this period and reversible neurocognitive deficits generally resolve by 3 months [12]. Consequently, the financial costs and patient burdens of prolonging follow-up cannot be ethically justified.

## Competing interests

DW is a consultant to Medtronic and Edwards, investigator for Edwards, Medtronic and Boston Scientific clinical studies and past proctor for Edwards. MV is a member of the Medtronic Asia-Pacific Surgical Advisory Board. No other author declares competing interests.

## Authors’ contributions

JPF and JFF were involved in the conception and design of study and drafting of the manuscript. AW, MS, EE, OT, WS and AB were involved in the design of the study and drafting of the manuscript. AC, JB, MV, DP, DW and PT were involved in the design of the study. All authors read and approved the final manuscript.

## Pre-publication history

The pre-publication history for this paper can be accessed here:

http://www.biomedcentral.com/1471-2261/14/45/prepub

## Supplementary Material

Additional file 1: Table S1 Comparison of Serological Markers of Neurological Injury. Click here for file

Additional file 2: Table S2 Comparison of Quality of Life (QoL) Measures. Click here for file
