# Supplementary material for: The silent and apparent neurological injury in transcatheter aortic valve implantation study (SANITY): concept, design and rationale
Source: BMC Cardiovasc Disord. 2014 Apr 5;14:45. doi: 10.1186/1471-2261-14-45 (PMC4021275; doi:10.1186/1471-2261-14-45)
Supplement: Additional file 1: Table S1 — Comparison of Serological Markers of Neurological Injury. [file 1471-2261-14-45-S1.docx]

**Supplementary Table 1: A comparison of Serological Markers of Neurological Injury**

| **Test** | **Function/Rational for Use** | **Strengths & Weaknesses** | **SANITY Study** |
| --- | --- | --- | --- |
| CK-BB | Involved in energy metabolism | Levels do not correlate with stroke severity | NO |
|  | of neural cells |  |  |
|  |  |  |  |
| Neuron-specfic enolase | A glycolytic enzyme involved in | Levels increase after acute brain injury | NO |
| (NSE) | anaerobic metabolism of glucose | Non-specific; levels increase after ANY injury |  |
|  |  | After acute stroke, levels do not correlate with function |  |
|  |  |  |  |
|  |  |  |  |
| Neurofilaments | Proteins integrated into neuronal | Relationship to infarct size & functional outcome unclear | NO |
| (NfH) | & axonal cytoskeleton |  |  |
|  |  |  |  |
| Tau | Microtubule-associated protein | Poor sensitivity for stroke; only high in 27-48% of patients | NO |
|  | stabilizes axonal cytoskeleton |  |  |
|  | & transport of vesicles to synapse |  |  |
|  |  |  |  |
| S100 proteins | Calcium-binding proteins involved | Best established marker of acute brain ischemia | YES |
|  | various cellular processes | Levels correlate with infarct size, clinical severity & |  |
|  |  | functional outcome |  |
|  |  | Presence in non-neural tissues like adipocytes, chondrocytes |  |
|  |  | & melanocytes reduce specificity |  |
|  |  |  |  |
| GFAP | Intermediate filament in astroglial | Levels correlate with infarct size, clinical severity & | YES |
|  | cytoskeleton; involved in repair of | functional outcome |  |
|  | neural tissue after injury | Subtypes exist specific to different tissues, which enhances |  |
|  |  | specificity |  |
|  |  | However, not yet validated in cardiac intervention patients |  |
