# Supplementary material for: The silent and apparent neurological injury in transcatheter aortic valve implantation study (SANITY): concept, design and rationale
Source: BMC Cardiovasc Disord. 2014 Apr 5;14:45. doi: 10.1186/1471-2261-14-45 (PMC4021275; doi:10.1186/1471-2261-14-45)
Supplement: Additional file 2: Table S2 — Comparison of Quality of Life (QoL) Measures. [file 1471-2261-14-45-S2.docx]

**Supplementary Table 2: A comparison of Quality of Life (QoL) Measures**

| **Instrument** | **# items** | **Strengths** | **Weaknesses** | **Used in** |
| --- | --- | --- | --- | --- |
| HEART-SPECIFIC HEALTH | | | | SANITY Study |
| Kansas City Cardiomyopathy Questionnaire (KCCQ) | 23 | Validated | Less often used than the MLHFQ | YES |
|  |  | Responsive to change |  |  |
|  |  | Prognostically valid |  |  |
|  |  | Validated in TAVI patients |  |  |
|  |  |  |  |  |
| Minnesota Living with Heart Failure Questionnaire (MLHFQ) | 21 | Most frequently used heart-specific measure | Not validated in TAVI patients | NO |
|  |  | Valid, responsive to change & reliable in | Less responsive than KCCQ |  |
|  |  | heart failure patients | Less flexible scale than KCCQ |  |
|  |  | Recommended by Oxford U. & UK Dept. Of Health | Higher cost |  |
|  |  |  |  |  |
| GENERAL HEALTH | | | |  |
| Medical Outcomes Study Short Form-36 (SF-36) | 36 | Validated across a wide range of conditions | Long & tedious to complete | NO |
|  |  | Most frequently-used general health measure | Floor & ceiling effects decrease responsiveness |  |
|  |  |  | Higher cost |  |
|  |  |  |  |  |
| Medical Outcomes Study Short Form-12 (SF-12) | 12 | Validated across a wide range of conditions | Fails to fully capture the psychological domain, | NO |
|  |  | Shorter & less tedious than the SF-36 | which is important given the association between |  |
|  |  |  | heart failure & depression |  |
|  |  |  | Floor & ceiling effects decrease responsiveness |  |
|  |  |  |  |  |
| European Quality of Life Questionnaire (EQ-5D) | 7 | Developed across 5 European countries | Not used as frequently as the SF-36 | YES |
|  |  | Short & easy to use |  |  |
|  |  | Specifically developed to be used in conjunction |  |  |
|  |  | with a disease-specific health measure |  |  |
|  |  | Good validity in heart failure patients |  |  |
|  |  | Recommended by Oxford U. & UK Dept. Of Health |  |  |
|  |  |  |  |  |
